# Genomic Analysis of Korean Patient With Microcephaly

**DOI:** 10.3389/fgene.2020.543528

**Published:** 2021-01-28

**Authors:** Jiwon Lee, Jong Eun Park, Chung Lee, Ah Reum Kim, Byung Joon Kim, Woong-Yang Park, Chang-Seok Ki, Jeehun Lee

**Affiliations:** ^1^Department of Pediatrics, Samsung Medical Center, Sungkyunkwan University School of Medicine, Seoul, South Korea; ^2^Department of Laboratory Medicine and Genetics, Hanyang University Guri Hospital, Hanyang University College of Medicine, Guri, South Korea; ^3^Samsung Genome Institute, Sungkyunkwan University School of Medicine, Seoul, South Korea; ^4^Department of Neurology, Samsung Medical Center, Sungkyunkwan University School of Medicine, Seoul, South Korea; ^5^Green Cross Genome, Yongin, South Korea

**Keywords:** microcephaly, whole exome sequencing (WES), chromosomal microarray, low-depth whole genome sequencing, Korea

## Abstract

Microcephaly is a prevalent phenotype in patients with neurodevelopmental problems, often with genetic causes. We comprehensively investigated the clinical phenotypes and genetic background of microcephaly in 40 Korean patients. We analyzed their clinical phenotypes and radiologic images and conducted whole exome sequencing (WES) and analysis of copy number variation (CNV). Infantile hypotonia and developmental delay were present in all patients. Thirty-four patients (85%) showed primary microcephaly. The diagnostic yield from the WES and CNV analyses was 47.5%. With WES, we detected pathogenic or likely pathogenic variants that were previously associated with microcephaly in 12 patients (30%); nine of these were *de novo* variants with autosomal dominant inheritance. Two unrelated patients had mutations in the *KMT2A* gene. In 10 other patients, we found mutations in the *GNB1, GNAO1, TCF4, ASXL1, SMC1A, VPS13B, ACTG1, EP300*, and *KMT2D* genes. Seven patients (17.5%) were diagnosed with pathogenic CNVs. Korean patients with microcephaly show a genetic spectrum that is different from that of patients with microcephaly of other ethnicities. WES along with CNV analysis represents an effective approach for diagnosis of the underlying causes of microcephaly.

## Introduction

Microcephaly is a prevalent phenotypic feature of patients with neurodevelopmental problems (Vargas et al., 2001; Abuelo, 2007). It is defined as an occipitofrontal circumference (OFC) lower than the third percentile or more than two standard deviations below the mean for sex, age, and ethnicity. Patients with microcephaly show various neurological manifestations, including psychomotor problems, delayed developments, and epilepsy, which are frequently accompanied by facial dysmorphism, skeletal anomalies, and congenital structural anomalies in the major organs (Vargas et al., 2001). Recognition of microcephaly can prompt clinicians to investigate its causes.

The fundamental size of the brain is determined by neuronal progenitor cells formed at conception and the cell divisions. During cell division, microcephaly may be caused by deficiency of neuropils, increased apoptosis of progenitor cells, or improper mitosis of these cells, which is defined as primary microcephaly (Gilmore and Walsh, 2013). Most primary microcephaly cases occur due to the failure of neurogenesis or destructive prenatal events associated with environmental or maternal conditions. In contrast, children with secondary microcephaly are born with normal head circumference (HC) and then show a progressive decrease in HC with age. Secondary microcephaly represents abnormal neuronal development after birth or a perinatal brain insult (Woods and Parker, 2013). Children with microcephaly have OFCs at the outer limits of the normal distribution and low for their age group. Both categories of microcephaly have been associated with various causative factors, including congenital infection, perinatal problems such as hypoxia or a maternal medical condition, or genetic causes (Abuelo, 2007), and the distinction between them helps clinicians to isolate the preferential etiologies. Additionally, accompanying anomalies or facial dysmorphism can provide clues to determine the exact cause of microcephaly (von der Hagen et al., 2014).

In previous studies, genetic causes of microcephaly showed an autosomal recessive inheritance pattern (Darvish et al., 2010; Sajid Hussain et al., 2013). With the development of next-generation sequencing, the genetic causes of neurodevelopmental disorders, including microcephaly, have been identified (Najmabadi et al., 2011; Hamdan et al., 2014; Thevenon et al., 2016). To date, only three studies have used whole exome sequencing (WES) to investigate genetic causes of microcephaly in microcephaly cohorts (Rump et al., 2016; Boonsawat et al., 2019; Shaheen et al., 2019). One study determined potential causative genes in 11 out of 38 patients who had highly heterogeneous clinical and radiologic phenotypes. It revealed that autosomal recessive disorders were highly prevalent among patients with microcephaly (Rump et al., 2016; Shaheen et al., 2019) identified potentially causal genetic variants in 104 out of 137 families. They found variants in novel and previously reported genes that were associated with microcephaly with established disease phenotypes. Additionally, the authors showed overlap in the genetic causes of microcephaly with genes underlying microcephalic primordial dwarfism. A recent comprehensive genetic analysis based on chromosomal microarray (CMA) and WES detected causative variants in 48.4% of the patients (30/62) and analyzed the differences in clinical severity and genetic variants between primary and secondary microcephaly (Boonsawat et al., 2019). Each of these studies covered different characteristics of race, consanguinity, and findings of brain imaging. However, there has been no study of an Asian population based exclusively on a microcephaly cohort.

Therefore, in this study, we aimed to comprehensively analyze the genomic and phenotypic features of Korean patients with microcephaly. We attempted to delineate the clinical features associated with the potential causative genes to contribute toward our understanding of the genetic features of patients with microcephaly.

## Materials and Methods

### Patients

**Figure 1:**
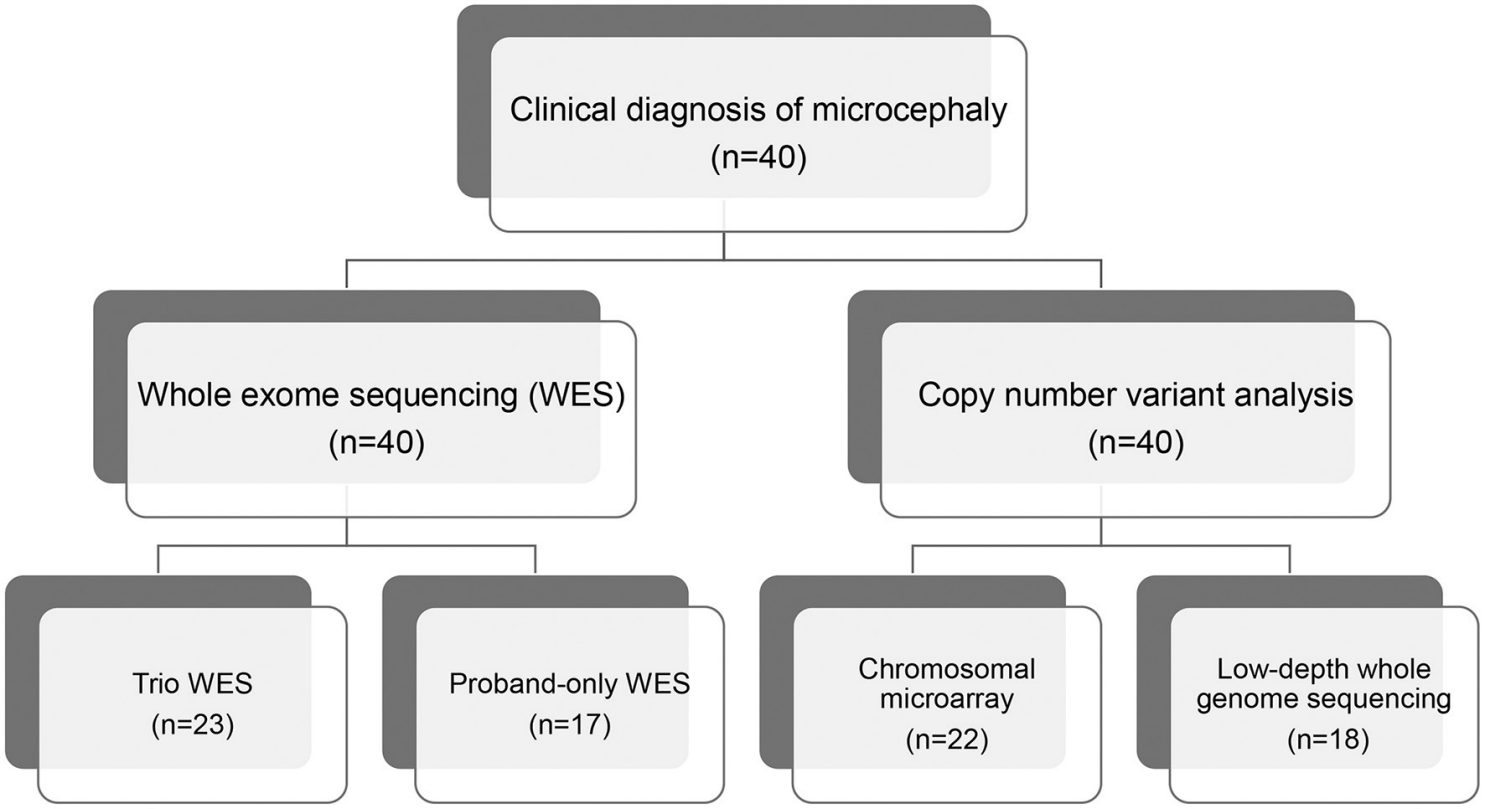
Flow chart of genomic analysis of microcephaly.

We recruited 40 pediatric patients with microcephaly, using a definition of microcephaly of OFC >2 standard deviations below the mean for same sex, age-matched Korean references, between January 2014 and December 2018 at the Samsung Medical Center in Seoul, Korea. All affected individuals were evaluated for clinical features and diagnosed with microcephaly by two pediatric neurologists. We excluded patients with microcephaly that resulted from cerebral insults related to defined perinatal problems such as congenital infection, teratogens, physical environment, or maternal medical conditions assessed via laboratory tests, brain imaging, and detailed medical history. We performed WES for all patients and CMA or low-depth whole genome sequencing (LD-WGS) to analyze copy number variation (CNV) (Figure 1). Among the patients that participated in this study, we performed trio WES for 23 patients whose parents' samples were available. The other 17 patients were subjected to proband-only WES. We measured weights, heights, and HCs in the same examination and evaluated concordance between the body gauges. Clinical phenotypes included the following: infantile hypotonia, facial dysmorphism, skeletal and organ anomalies, hearing loss, developmental delay/cognitive impairment, seizure, and affected epilepsy syndrome. Development and cognitive function were assessed using the Bayley Scales of Infant Development (BSID-III) or the Wechsler Scale of Intelligence for children (WISC-V), depending on the age of the patient. If these scales were not available, we estimated developmental stage or cognitive function based on the clinical status assessed by two pediatric neurologists using the same standards.

### WES

Genomic DNA was extracted from peripheral blood leukocytes using a Wizard Genomic DNA Purification Kit following the manufacturer's instructions (Promega, Madison, WI, USA). SureSelect Human All Exon V5 (Agilent Technologies, Santa Clara, CA, USA) was used for library preparation, and sequencing was performed on an Illumina HiSeq2500 platform (Illumina Inc., San Diego, CA, USA) to generate 2 × 100-bp paired-end reads. Sequence reads were aligned against the Human Reference Genome build GRCh37 using BWA 0.7.12; duplicated reads were marked with Picard Tools 1.130. Local alignment, base quality recalibration, and variant calling were conducted using the Genome Analysis Tool Kit v3.4.0, and annotation and variant effect prediction were performed with SnpEff v4.1g.

### Variant Filtering Steps and Data Analysis

Variants were filtered and prioritized using a four-step strategy to generate a short candidate variant list for experimental validation (Supplementary Figure 1). Initially, we removed variants with <10× coverage. However, before removing <10× variants, ClinVar (https://www.ncbi.nlm.nih.gov/clinvar/) and Human Gene Mutation Database (HGMD, http://www.hgmd.org/) were checked for any previously reported pathogenic or likely pathogenic variants, and such variants were not filtered out regardless of whether they were <10×. Next, variants were limited to those with low population frequency. The minor allele frequency (MAF) threshold was carefully chosen and variants with an MAF ≥ 1% in the Genome Aggregation Database (gnomAD) (http://gnomad.broadinstitute.org/) or the Korean Reference Genome Database (KRGDB) (http://coda.nih.go.kr/coda/KRGDB/index.jsp) were removed. The third step was to prioritize variants causing missense, non-sense, frameshifts, and in-frame insertions/deletion variants, or changes affecting consensus splice site sequences. Finally, we performed a gene-specific analysis with an *in silico* gene panel composed of 885 genes, for filtering selected phenotype traits based on the Human Phenotype Ontology (HPO)-terms for Microcephaly (HP:0000252) or Online Mendelian Inheritance in Man (OMIM) microcephaly phenotype genes (Supplementary Table 1). To delineate candidate genetic variants, an additional allele analysis was performed under the following conditions: (1) triplicate data with no pathogenic variants (PVs) nor likely pathogenic variants (LPVs), (2) *de novo*, compound heterozygous, homozygous, or hemizygous variants, (3) ≤ 2 alleles in gnomAD or ≤ 8 alleles if recessive, (4) a CADD score of ~15 or higher and all deleterious predictions in SIFT (http://sift.jcvi.org), PolyPhen2 (http://genetics.bwh.harvard.edu/pph2), and MutationTaster (http://mutationtaster.org/) if missense variants, (5) affected genes with data from animal models and/or functional studies suggesting neurodevelopmental roles.

### CMA and LD-WGS

CMA was performed using an Illumina HumanCytoSNP-12 platform (Illumina, San Diego, CA, USA). The array employs both Copy Number Changes (CNC) and Single Nucleotide Polymorphism (SNP) probes on a whole genome array with a 30-Kb resolution. A higher resolution was used for all regions known to be associated with balanced and unbalanced structural variants. The data were analyzed in the Illumina KaryoStudio version 1.4.3.0 (Illumina, San Diego, CA, USA) and were reported using NCBI human genome build 37.1 (hg19) following ISCN nomenclature.

The LD-WGS-based CNV analysis was performed by GC Genome (Yongin, Korea). Briefly, genomic DNA was extracted from peripheral blood leukocytes, sheared to a target size of 250 bp, and sequenced on a NextSeq 500 platform (Illumina, CA, USA) in a 75-bp single-read mode. CNV calling for 3.1 million sequence reads was performed using the DNAcopy software v1.38. Using the target-specific reference mapping ratio values for the target sample and the normal controls, we calculated the copy number status of each targeted sample. The February 2009 Human reference sequence (GRCh37/hg19) was used for genome assembly.

### Interpretation of Candidate Genetic Variants by WES and CNVs by CMA or LD-WGS

Candidate variants were classified according to the standards and guidelines of the American College of Medical Genetics and Genomics (ACMG) and Association for Molecular Pathology (AMP) (Richards et al., 2015). These guidelines recommend classifying variants into five categories: PV, LPV, variant of uncertain significance (VUS), likely benign variant (LBV), and benign variant (BV) based on combined lines of weighted evidence, including population, computational, functional, segregation, *de novo*, allelic, and other data. To assess the frequency of a variant in a control or general population, we used the KRGDB, which consists of publicly available race-matched control data from WGS of 1100 Korean individuals, as well as other public databases such as the 1,000 Genomes Project database, ExAC database, and gnomAD. A primary literature review was conducted using various sources cited in the HGMD professional version (release 2018.02), ClinVar, and PubMed, to determine the potential pathogenicity of all identified variants. Various *in silico* tools, including SIFT, PolyPhen2, and MutationTaster, all of which use missense prediction algorithms, were used to determine the predicted impact of a missense change.

To interpret CNVs, we used DGV (http://dgv.tcag.ca/dgv/app/home), DECIPHER (https://decipher.sanger.ac.uk/), and dbVar (https://www.ncbi.nlm.nih.gov/dbvar/) databases as well as OMIM (https://www.omim.org/). Additionally, the clinical findings were compared with those reported in the literature.

### Confirmation and Validation of Candidate Variants

Candidate variants identified in WES data were confirmed using standard PCR and Sanger sequencing methods. Primer sequences are available upon request. Sequence data were aligned to the reference sequence using the Sequencher software (Gene Codes Corporation, Ann Arbor, MI, USA).

## Results

### Clinical Phenotypes of Patients

Among 40 patients (female:male = 22:18) with microcephaly, 34 patients presented with primary microcephaly at birth and the remaining six were identified as cases of secondary microcephaly based on the gradual development of a small HC after birth (Table 1). With proportionate microcephaly identified in 15 patients (37.5%), all body gauges were below the sex and age-matched 3rd percentile value. The other 25 patients (62.5%) showed disproportionate microcephaly, with height and/or weight in the normal range for sex and age. All patients with short stature (*n* = 26) displayed type 3 failure to thrive during their infancy. Common features observed in all patients were infantile hypotonia and developmental delay. The average age at the time of participation in this study was 5.28 ± 4.60 years. Thirty-two patients showed noticeable developmental delay or intellectual disability. Of the remaining patients, one showed mild intellectual disability and seven displayed a delay (5 months below age) in reaching developmental milestones. Patients of a preschool age (*n* = 25) also lagged in achieving developmental milestones, and eight patients of a schoolgoing age received special education. Four patients were treated for autism spectrum disorder (*n* = 3) or behavioral disorder (*n* = 1) in a neuropsychiatric clinic. Seven patients showed frequent dystonia or other involuntary movements like chorea, athetoid movements, or tremor.

**Table 1:** Clinical phenotypes of 40 patients with microcephaly.

**Presentation time**	***N* (%)**
Primary microcephaly	34 (85.0)
Secondary microcephaly	6 (15.0)
**Proportion to other body gauges**
Proportionate	15 (37.5)
Disproportionate	25 (62.5)
**Infantile hypotonia**	40 (100)
**Delayed development**	40 (100)
**Neurocognitive function**
Intellectual disability	11 (27.5)
Intellectual disability with autism spectrum disorder	3 (7.5)
Intellectual disability with behavioral disorder	1 (2.5)
Impossible to define the intelligence^*^	25 (62.5)
**Movement disorder**	7 (17.5)
**Dysmorphic features of face**	28 (70.0)
Definite dysmorphism	17 (42.5)
Vague faces	11 (27.5)
**Accompanied anomaly**	17 (42.5)
Heart anomaly	8 (20.0)
Skeletal anomaly	4 (10.0)
Cleft lip and/or palate	5 (12.5)
Sensorineural hearing loss	3 (7.5)
Congenital cataract/Microphthalmia	2 (5.2)
Others^†^	5 (12.5)
**MRI findings**
Microcephaly vera	34 (85.0)
With holoprosencephaly	1 (2.5)
With agenesis or dysgenesis of corpus callosum	3 (7.5)
With subependymal heterotopia and simplified gyrus	1 (2.5)
With hypoplasia of pons and cerebellum	1 (2.5)
**Presence of seizure**	27 (67.5)
Intractable epilepsy	11 (27.5)
Controlled epilepsy	15 (37.5)
Seizure with febrile illness	1 (2.5)
**Epilepsy diagnosis**	27 (67.5)
Lennox-Gastaut syndrome (LGS)	6 (15.0)
West syndrome	1 (2.5)
Early onset epileptic encephalopathy, not LGS	2 (5.0)
Early onset intractable epilepsy	5 (12.5)

* *Patients were too young or severely retarded to assess their intelligence*.

*Other anomalies included diaphragmatic hernia (n = 1), syringomyelia (n = 1), biliary atresia (n = 1), ambiguous genitalia (n = 1), and gastro-intestinal malrotation (n = 1)*.

*LGS included that neonatal seizure (n = 1) or West syndrome (n = 3) evolving to LGS*.

Seventeen patients (42.5%) showed various accompanying anomalies, including congenital heart anomaly (*n* = 8), skeletal anomaly (*n* = 4), and cleft lip and/or palate (*n* = 5) (Table 1). Three patients developed sensorineural hearing loss without a discernible etiology. Dysmorphic features were evident in more than half of the patients (*n* = 28, 70%); these features were clear in 17 patients (42.5%) and notably vague in 11 patients (27.5%). Twenty-seven patients (67.5%) had seizures. One of these patients had only one episode of seizure provoked by febrile illness. Among the 26 patients with epilepsy, 11 showed intractable epilepsy, with frequent seizures despite multiple anti-epileptic medications. There were eight patients diagnosed with epileptic encephalopathy, including six patients with Lennox-Gastaut syndrome (LGS). Three of the patients with LGS showed infantile spasms, and one patient had a neonatal seizure.

Brain MRIs were performed in all patients, and abnormal structural lesions were found in six patients. Three patients had agenesis or hypoplasia of the corpus callosum with microcephaly. In another three patients, holoprosencephaly, subependymal heterotopia, and hypoplasia of the pons and cerebellum were found, respectively. Thirty-four patients showed normal brain structure using MRI. Further details of all patients can be found in Supplementary Table 2.

### Genetic Evaluation of Patients

A molecular diagnosis was established in 19 patients (47.5%) from 39 families. In 12 patients, including one sibling pair (30%), 11 PVs or LPVs were identified by WES (Table 2). The inheritance patterns of these mutations were autosomal dominant (*n* = 9), autosomal recessive (*n* = 1), and X-linked dominant (*n* = 1). Another seven patients (17.5%) showed a pathogenic CNV by CMA or LD-WGS (Table 3). Using WES, PVs or LPVs were detected in 10 genes previously associated with microcephaly: *GNB1, GNAO1, TCF4, ASXL1, SMC1A, KMT2A, VPS13B, ACTG1, EP300*, and *KMT2D*. Five PVs or LPVs were novel variants, and nine PVs or LPVs were *de novo* variants. Identified variants except BV and LBV are described in Supplementary Table 3.

**Table 2:** Clinical characteristics of 12 patients having pathogenic or likely pathogenic variants via whole exome sequencing.

**No**.	**Sex**	**Age**	**Onset time**	**Proportion**	**Genetic variants**								**Neurocognitive function**	**Dysmorphic face**	**Accompanying anomalies**	**Epilepsy**	**Other features**	**Disorder**
					**Gene**	**Inheritance**	**Zygosity**	***De novo***	**Refseq**	**Nucleotide**	**Amino acid change**	**2015 ACMG**						
M-011	F	9y10m	PM	P	*GNB1*	AD	Het	+	NM_002074.4	c.284T>C	p.(Leu95Pro)	LPV	Uncheckable	Present	Arthrogryposis, club foot	IS → LGS		Epileptic encephalopathy
M-020	M	4y7m	SM	P	*GNAO1*	AD	Het	+	NM_020988.2	c.607G>A	p.(Gly203Arg)	LPV	Uncheckable	Present	None	EIEE	Intractable dystonia, DCMP	Epileptic encephalopathy
M-036	M	4y9m	PM	DP	*TCF4*	AD	Het	+	NM_001083962.1	c.1813C>T	p.(Gln605Ter)	PV	K-ABC 52 ± 9	None	None	None	Complex febrile seizure	Pitt-Hopkins syndrome
													SQ 29					
M-039	F	10m	PM	DP	*ASXL1*	AD	Het	+	NM_015338.5	c.3115C>T^†^	p.(Gln1039Ter)	PV	Uncheckable	Present	Cleft palate, choanal atresia, CHD	Infantile onset epilepsy (under control)		Bohring-Opitz syndrome
M-055-P	M	4y1m	PM	P	*SMC1A*	XLD	Hemi	–	NM_006306.3	c.2368C>T	p.(Arg790Trp)	LPV	Uncheckable	Present	Cleft palate, CHD	Infantile onset epilepsy (under control)	Hearing loss	Cornelia De Lange syndrome
M-055-S	M	1y5m	PM	P	*SMC1A*	XLD	Hemi	–	NM_006306.3	c.2368C>T	p.(Arg790Trp)	LPV	Uncheckable	Present	Cleft palate, GI malrotation	Infantile onset epilepsy (under control)	Hearing loss	Cornelia De Lange syndrome
M-056	M	7y2m	PM	P	*KMT2A*	AD	Het	NA	NM_001197104.1	c.10217C>G^†^	p.(Ser3406Ter)	LPV	FSIQ: 51	Present	Skeletal anomaly	None	Severe constipation	Wiedemann-Steiner syndrome
M-073	F	4y11m	SM	DP	*KMT2A*	AD	Het	+	NM_001197104.1	c.2552_2553del^l^^†^	p.(Lys851ArgfsTer14)	PV	Uncheckable	Present	None	None	Cyclic vomiting	Wiedemann-Steiner syndrome
M-131	F	4y5m	PM	DP	*ACTG1*	AD	Het	+	NM_001199954.2	c.628C>T	p.(Arg210Cys)	LPV	Uncheckable	Present	Cleft lip	None		Baraitser-Winter syndrome
M-143	M	2y8m	PM	P	*VPS13B*	AR	Het	–	NM_017890.4	c.1950C>A^†^	p.(Cys650Ter)	LPV	FSIQ: 41	None	None	None		Cohen syndrome
					*VPS13B*	AR	Het	–	NM_017890.4	c.9981G>A^†^	p.(Trp3327Ter)	PV	ASD					
M-145	F	1y1m	PM	DP	*EP300*	AD	Het	+	NM_001429.3	c.1453del^†^	p.(Gln485ArgfsTer22)	PV	Uncheckable	Present	None	None		Rubinstein-Taybi syndrome
M-149	M	6y	PM	P	*KMT2D*	AD	Het	NA	NM_003482.3	c.7228C>T	p.(Arg2410Ter)	PV	Uncheckable	Present	Cleft palate, CHD, diaphragmatic hernia	Intractable epilepsy		Kabuki syndrome

*AD, autosomal dominant; ASD, autism spectrum disorder; AR, autosomal recessive; CHD, congenital heart disease; DP, disproportionate; EIEE, early-infantile epileptic encephalopathy; FSIQ, full scale intelligence quotient; GI, gastrointestinal; IS, infantile spasms; ISCN, International System for Human Cytogenetic Nomenclature; IQ, intelligence quotient; LGS, Lennox-Gastaut syndrome; MICPCH, microcephaly with pontine and cerebellar hypoplasia; P, proportionate; PM, primary microcephaly; SM, secondary microcephaly; SQ, social quotient; XLD, X-linked domiant*.

† *Novel variant (n = 5)*.

**Table 3:** Clinical characteristics of seven patients having pathogenic copy number variants.

**No**.	**Sex**	**Age**	**Onset time**	**Proportion**	**Genetic variants**		**Neurocognitive function**	**Dysmorphic face**	**Accompanying anomalies**	**Epilepsy**	**Other features**	**Disorder**
					**ISCN description**	**Size**						
M-004	M	20 y	PM	P	arr 7q11.21(69,404,786–70,206,198) ×1	801 Kb	FSIQ: 34, ASD	Present	None	Intractable epilepsy		AUTS2 syndrome
M-012	F	9y7m	PM	P	arr 2q24.3(166,340,583–166,904,859) ×1	564 Kb	Uncheckable	None	None	Dravet syndrome		Dravet syndrome
M-017	F	4y2m	PM	DP	arr 9q34.3(138,225,001–141,015,001) ×1	2,790 Kb	IQ: uncheckable, SQ: 24	Vague	CHD	IS → LGS		Kleefstra syndrome
M-048	F	1y4m	PM	P	arr 16p11.2p13.3 (5,805,001–34,230,001) ×3	28.43 Mb	Uncheckable	Vague	CHD	Neonatal seizure → LGS		16p11.2 deletion syndrome
M-074	M	9y8m	PM	P	arr 6q25.3(157,075,546–157,443,054) ×3	368 Kb	IQ: uncheckable, ASD	Present	Cleft palate, cryptorchidism	Controlled epilepsy		Coffin-Siris syndrome
M-075	F	5m	PM	DP	arr Xp11.4p11.3(41,150,139–43,976,458) ×1	2.8 Mb	Uncheckable	Present	None	None	Hypoplasia of cerebellum and pons	MICPCH
M-116	M	4y9m	SM	DP	arr 2q23.1(148,710,290–149,021,799) ×1	312 Kb	IQ: uncheckable, SQ: 55, ASD	Present	None	None		Intellectual disability with ASD

*ASD, autism spectrum disorder; CHD, congenital heart disease; DP, disproportionate; FSIQ, full scale intelligence quotient; IS, infantile spasms; ISCN, International System for Human Cytogenetic Nomenclature; IQ, intelligence quotient; LGS, Lennox-Gastaut syndrome; MICPCH, microcephaly with pontine and cerebellar hypoplasia; P, proportionate; PM, primary microcephaly; SM, secondary microcephaly; SQ, social quotient*.

### Clinical Phenotypes of the Patients With a PV/LPV Determined by WES

There was a 9-year-old girl (M-011) with an LPV in the *GNB1* gene. She presented with infantile spasms at the age of 4 months and was treated with anti-epileptic medications. Her epileptic spasms disappeared during infancy, but she failed to reach developmental milestones. Gradually, different types of seizures and involuntary movements developed, despite administration of a number of anti-epileptic medications. A *de novo* LPV (c.284T>C;p.Leu95Pro) that was absent from the control databases was identified in the *GNB1* gene. This c.284T>C variant was previously associated with *de novo* severe neurodevelopmental disability, hypotonia, and seizures (Petrovski et al., 2016).

In a 4-year-old boy (M-020) suffering from intractable dystonia with severe failure to thrive and profound psychomotor retardation, a *de novo* LPV (c.607G>A;p.Gly203Arg) that was absent from the control databases was identified in the *GNAO1* gene. A *de novo* c.607G>A change in *GNAO1* has been previously reported many times in patients with epileptic encephalopathy, suggesting that it is a mutation hotspot (Arya et al., 2017).

In patient M-039, a PV (c.3115C>T;p.Gln1039Ter) of the *ASXL1* gene was identified. She was admitted to the neonatal intensive care unit for congenital hypotonia. As her respiration was unstable and weak, a tracheostomy was performed and a home ventilator support was provided for her. She showed severe feeding problems and extreme failure to thrive. Additionally, she suffered from intractable epilepsy and severe dystonia. The c.3115C>T variant in *ASXL1* was absent in the control population and occurred *de novo*. This c.3115C>T variant was previously reported mainly as a somatic variant in patients with myelodysplastic syndrome (Wang et al., 2013), but no germline variant has been reported.

In the *SMC1A* gene, known as a causative gene of Cornelia de Lange syndrome (Ansari et al., 2014), an LPV (c.2368C>T;p.Arg790Trp) was identified in a male sibling pair (M-055-P, M-055-S) clinically suspected of Cornelia de Lange syndrome. The pair showed distinctive facial features, namely long eyelashes, synophrys, small nose, wide nasal bridge, low-set ears, and small chin. They could not speak meaningful words and showed severe growth retardation in not only HC, but also height and weight. The c.2368C>T variant in *SMC1A* was hemizygous. The patients' mother had this variant; in her case, it was heterozygous. There were two unaffected maternal uncles, who were unmarried. They displayed normal intelligence and facial features, and were not tested for the variant.

### Clinical Phenotypes of Patients With a Pathogenic CNV

Of the 40 patients, 7 (17.5%) were found to have a pathogenic CNV by CMA or LD-WGS (Table 3). The size of duplication or deletion region was between 312 Kb and 28.43 Mb.

Patient M-004 presented with severe intellectual disability, autistic behaviors, epilepsy, and dysmorphic facial features, and showed an 801-Kb deletion at 7q11.21. This region contains the *AUTS2* gene, and the deletion of the *AUTS2* gene has been reported to be associated with autism, intellectual disability, short stature, and microcephaly (Beunders et al., 2013).

M-017 presented with primary microcephaly and congenital heart disease, and showed a 2,790-Kb deletion at 9q34.3. This region was previously associated with 9q subtelomeric syndrome characterized by severe developmental delay, microcephaly, congenital heart disease, and seizure (Stewart and Kleefstra, 2007).

M-048 presented with primary microcephaly and congenital heart disease, and displayed a 28.43-Mb duplication at 16p13.3p11.2. This region was registered at OMIM as being associated with chromosome 16p11.2 duplication syndrome (MIM 614671). Patients with a 16p11.2 duplication have been reported to exhibit motor delays, congenital anomalies, seizures, and microcephaly (Shinawi et al., 2010).

M-074 presented with primary microcephaly, epilepsy, and cleft palate, and carried a 368-Kb duplication at 6q25.3. This region contains the *ARID1B* gene, whose mutation is responsible for Coffin-Siris syndrome. This syndrome is characterized by mental retardation and associated with coarse facial features, hypertrichosis, sparse scalp hair, and hypoplastic or absent fifth fingernails or toenails. Patients with a *ARID1B* duplication have been reported to show moderate developmental delays, hypotonia, and high-arched palate (Hoyer et al., 2012).

**Figure 2:**
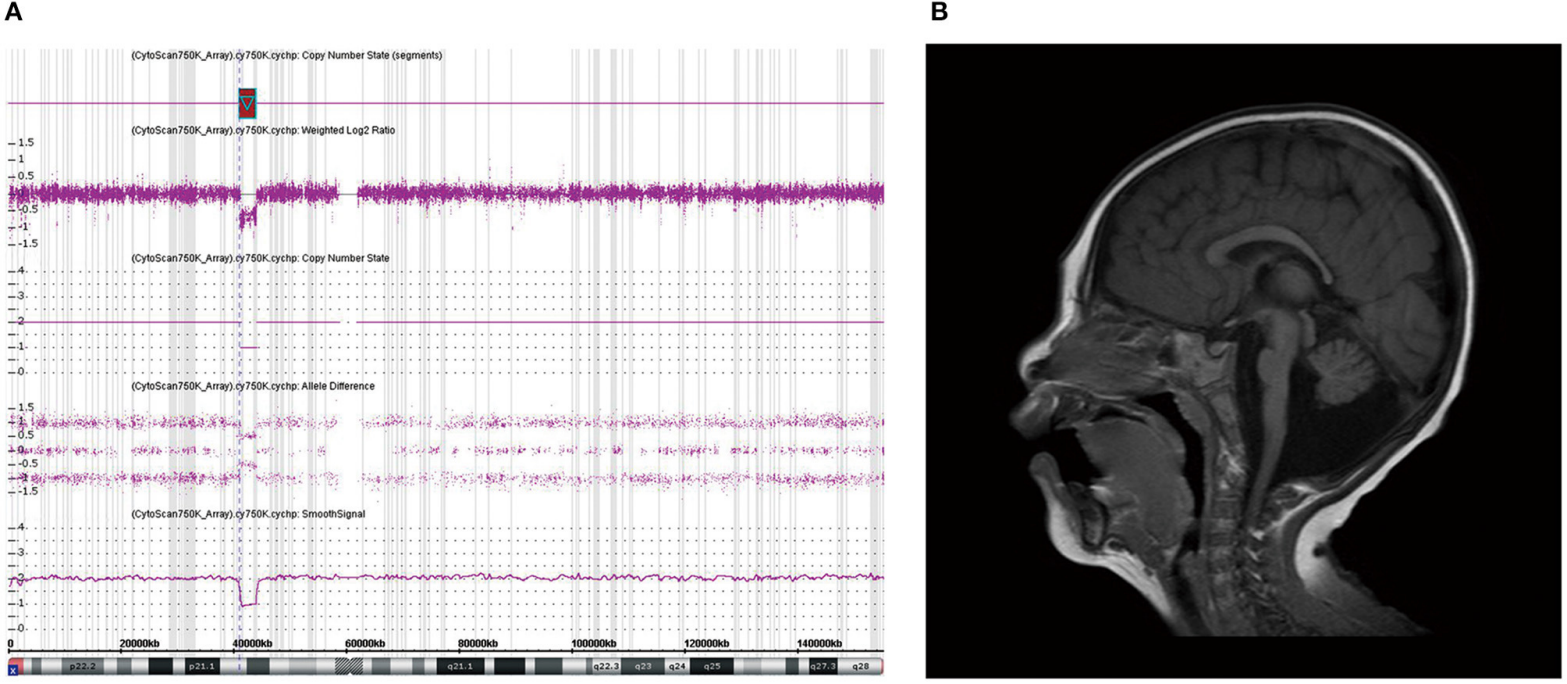
Chromosomal microarray (CMA) and brain magnetic resonance imaging (MRI) of patient M-075. **(a)** CMA showing a 2.8-Mb deletion at Xp11.4p11.3 containing the *CASK* gene. **(b)** Brain MRI of patient (M-075) demonstrating the microcephaly with pontine and cerebellar hypoplasia in the T2-weighted sagittal view.

A girl (M-075) showing microcephaly with pontine and cerebellar hypoplasia evidenced by brain MRI was found to have a 2.8-Mb deletion at Xp11.4p11.3 (Figure 2). This region contains the *CASK* gene, whose deletion has been associated with mental retardation and microcephaly with disproportionate pontine and cerebellar hypoplasia in females (Hayashi et al., 2012).

M-116 presented with intellectual disability and autistic features, midline hypoplasia of the face, hypertrichosis, and bush eyebrows, and showed a 312-Kb deletion at 2q23.1. This region contains the *MBD5* gene, whose deletion has been associated with intellectual disability, seizures, significant speech impairment, and behavioral problems (Bonnet et al., 2013).

### Additional Candidate Microcephaly Genes

An allele search in familial trio exome data revealed four additional candidate genes (Table 4). In a patient (M-001) with primary microcephaly, we found a *de novo* variant (c.2123A>C;p.Tyr708Ser) in the *JARID2* gene. *JARID2* (jumonji and AT-rich interaction domain containing 2) is highly homologous to mouse Jumonji (Jmj). In mice, Jmj is required for normal morphogenesis of the neural tube (Takeuchi et al., 1995). This c.2123A>C variant in *JARID2* was absent from the control databases.

**Table 4:** Clinical characteristics of four patients having a variant of additional candidate genes.

**No**.	**Sex**	**Age**	**Onset time**	**Proportion**	**Genetic variants**								**Neurocognitive function**	**Dysmorphic face**	**Accompanying anomalies**	**Epilepsy**	**Other features**	**Disorder**
					**Gene**	**Inheritance**	**Zygosity**	***De novo***	**Refseq**	**Nucleotide**	**Amino acid change**	**2015 ACMG**						
M-001	F	11m	PM	P	*JARID2*	AD	Het	+	NM_004973.3	c.2123A>C	p.(Tyr708Ser)	VUS	Uncheckable	Vague	Holoprosen-cephaly	IS → LGS	None	Epileptic encephalopathy
M-100	M	5y11m	PM	P	*RFX7*	AD	Het	+	NM_022841.6	c.2727_2730dup	p.(Gly911PhefsTer58)	VUS	Uncheckable	Vague	None	None	None	Intellectual disability
M-118	M	4y4m	PM	DP	*LMNB1*	AD	Het	+	NM_005573.3	c.1091T>C	p.(Leu364Pro)	VUS	Uncheckable	Vague	Subependymal heterotopia	LGS	None	Epileptic encephalopathy
M-122	M	1y5m	PM	P	*HPRT1*	XLD	Hemi	–	NM_000194.3	c.151C>T	p.(Arg51Ter)	VUS	Uncheckable	Vague	Valgus of ankle	None	Café au lait spot	Neurofibro-matosis

*2015 ACMG, 2015 American College of Medical Genetics and Genomics and Association for Molecular Pathology guideline; AD, autosomal dominant; DP, disproportionate; Hemi, hemizygous; Het, heterozygous; IS, infantile spasms; LGS, Lennox-Gastuat syndrome; P, proportionate; PM, primary microcephaly; SQ, social quotient; VUS, variant of uncertain significance; XLD, X-linked dominant*.

*RFX7* (regulatory factor X7) is a member of the regulatory factor X (RFX) family of transcription factors (Aftab et al., 2008). RFX7 is required for the formation of cilia in the neural tube (Manojlovic et al., 2014). In one patient (M-100), we found a *de novo* variant (c.2727_2730dup;p.Gly911PhefsTer58) in the *RFX7* gene that was absent from the control databases.

*LMNB1* (lamin B1) encodes one of the two B-type lamin proteins. Lamin B1, a key component of the nuclear lamina, plays an important role in brain development (Mahajani et al., 2017). A heterozygous tandem genomic duplication of *LMNB1* is known to be the cause of autosomal dominant adult-onset demyelinating leukodystrophy. In one patient (M-118), we found a *de novo* variant (c.1091T>C;p.Leu364Pro) in this gene that was absent from the control databases.

*HPRT1* (hypoxanthine-guanine phosphoribosyltransferase 1) encodes hypoxanthine-guanine phosphoribosyl transferase, an enzyme involved in purine metabolism. An *HPRT1* gene mutation is the cause of Lesch-Nyhan syndrome, which is an X-linked recessive disorder (Gibbs and Caskey, 1987). In one patient (M-122), we identified a hemizygous variant (c.151C>T;p.Arg51Ter) that was inherited from his mother. This c.151C>T variant was absent from the control databases and was previously reported in a Japanese patient with Lesch-Nyhan syndrome (Fujimori et al., 1990). The patient in this study did not have the clinical characteristics of that syndrome.

## Discussion

This study used WES and CNV analyses to identify genetic causes of microcephaly in Korean patients. The overall diagnostic rate was 47.5%, with 12 cases (30%) identified by using WES and 7 cases identified by using CNV analysis. Nine variants among all the variants found by using WES, including five novel ones, were *de novo* and exhibited autosomal dominant traits. Of the 40 patients included in this study, 34 patients (85%) had primary microcephaly. Six showed various structural anomalies as evidenced by brain MRIs, whereas the others showed normal brain structures despite microcephaly. Hypotonia and developmental delay were the most frequent accompanying symptoms. Seizure was the next most frequent symptom (*n* = 27, 65%), and 11 patients had intractable seizures.

Microcephaly can present as non-syndromic or with various hereditary syndromic features (von der Hagen et al., 2014). According to the OMIM database, there are reportedly over 900 OMIM phenotypes and genes related to microcephaly. In the present study, the diagnostic yield of WES (PVs and LPVs) was 30% and was comparable to that found in previous reports of genetic causes of microcephaly (Rump et al., 2016; Boonsawat et al., 2019; Shaheen et al., 2019). In general, the diagnostic yield of WES in neurodevelopmental disorders ranges from 17.5 to 29% (Vissers et al., 2017; Marques Matos et al., 2019). The yield depends on several factors such as phenotypic distinctiveness of the disease, genes associated with the phenotype, sequence read depth, bioinformatics filtering, and/or level of clinical medical review (Farwell et al., 2015; Di Resta and Ferrari, 2018; Prodan Zitnik et al., 2018; Trinh et al., 2019). Some neurological disorders are more closely associated with muscle disease, ataxia, and epilepsy, which are known to have heterogeneous genetic causes (Farwell et al., 2015; Marques Matos et al., 2019). Thus, WES should be the first-line test for complex diseases presumed to have extensive genetic causes, based on considerations of diagnostic yield and economic cost (Vissers et al., 2017; Marques Matos et al., 2019). Similar to those identified in previous reports, the variants found in this study were highly diverse. This study reconfirms the utility of WES in determination of the genetic causes of microcephaly.

In this study, the CNV analysis increased the diagnostic rate from 30 to 47.5%. This rate was similar to that reported in a recent study by Boonsawat et al. (2019). The diagnostic rate of pathogenic CNVs in neurodevelopmental disorders, including primary microcephaly, has been reported to be 13.2–20.8% (Shoukier et al., 2013; Jang et al., 2019). According to a previous study, combined WES and CNV analyses enable better diagnosis of rare neurological disorders, with a 47.8% diagnostic rate, compared to that observed with WES alone (32.7%) (Jiao et al., 2019), which is consistent with the results of this study. A recent study showed 11 definite and 7 probable CNVs in 53 patients with primary microcephaly (Tsoutsou et al., 2017). Therefore, WES with CNV analysis may be the most effective approach to diagnose the underlying causes of microcephaly. A diagnosis of microcephaly via WES and CNV analysis can help to determine the genetic mechanism of the disease and predict the prognosis. Additionally, it may provide information to patients for future reproductive decisions and genetic counseling, including opportunities for targeted therapies.

In the present study, most of the detected variants showed an autosomal dominant inheritance pattern. Two variants were associated with autosomal recessive and X-linked dominant inheritance, respectively. Previous studies dealt with congenital microcephaly in consanguineous families and mainly focused on neurodevelopmental defects in the fetal period (Darvish et al., 2010; Sajid Hussain et al., 2013; Shaheen et al., 2019). They classified cases of microcephaly as autosomal recessive primary microcephaly (MCPH), and 18 loci in MCPH genes have been revealed (Jayaraman et al., 2018). In our patients, no mutations were identified in *MCPH1, WDR62, CDK5RAP2, CEP152, ASPM, CENPJ, STIL, CEP63, CEP135, CASC5, PHC1, CDK6, CENPE, SASS6, MFSD2A, ANKLE2, CIT*, or *WDFY3* genes. Our results suggest that autosomal dominant disorders are highly prevalent among Korean patients with microcephaly. The microcephaly-associated genes identified in this study were diverse, and no genes were found to be predominant causes of microcephaly. This suggests that various genes may lead to microcephaly, as reported previously (Shaheen et al., 2019). These genes are involved in neuronal myelination, neurotransmission, or regulation of neuronal excitability, rather than being involved in centrosome-related neurogenesis and DNA damage repair process (Woods and Parker, 2013).

Among 19 patients who were established their genetic diagnosis in this study, 14 patients were concluded as syndromic microcephaly. Syndromic microcephaly can occur in both primary and secondary microcephaly and most are accompanied by other organ anomalies (von der Hagen et al., 2014). Through these characteristic phenotypes, including other organ anomalies, dysmorphism, laboratory findings, we can predict a putative cause of the patient with microcephaly indirectly. However, even with syndromic microcephaly, it is not easy to approach the genetic diagnosis only with clinical information which the patient has. Some of the patients with syndromic microcephaly confirmed genetically in this study also had no other organ anomalies, but only had intellectual disabilities or vague dysmorphism. For this reason, it was difficult to conclude the genetic diagnosis with only developmental delay and small head circumference. In the other two studies of microcephaly that included syndromic and non-syndromic microcephaly, various genetic findings were found through WES and CMA similar to our study (Rump et al., 2016; Boonsawat et al., 2019). On the other hand, the results of study on non-syndromic microcephaly by Shaheen et al. (2019) showed different genetic spectrum of microcephaly.

In this study, 34 patients showed normal brain structures despite a small brain size. In the six patients (15%) with structural abnormalities observed by brain MRI, only one patient was found to have a causative genetic mutation in the *CASK* gene. The *CASK* gene encodes a membrane-associated guanylate kinase and is involved in neurotransmitter regulation, axon branching, and dendritic outgrowth. The patient with this gene variant showed a wide range of X-linked intellectual disabilities, including microcephaly with pontine and cerebellar hypoplasia (MICPCH) syndrome, nystagmus, dysmorphic faces, and hypotonia (Hayashi et al., 2017). As this is located on the X chromosome, most cases of *CASK* mutation are exhibited by females, and affected males are very rare and show severe manifestations (Cristofoli et al., 2018). We found a *CASK* mutation through CMA in a female patient with MICPCH syndrome. She was born at term without perinatal complications. Her HC at birth was 32.2 cm, and the fourth ventricle was extensively dilated according to a brain ultrasonogram performed on the second day of birth. She showed poor growth in all body indices and dysmorphic facial features such as arched eyebrows, midline hypoplasia, long philtrum, and micrognathia. Her brain MRI findings were consistent with the criteria for microcephaly and pontocerebellar hypoplasia, and her achievement of developmental milestones being assessed at 1 year of age showed at the 5-month level (Figure 2).

Racially homogeneous Korean patients with microcephaly, with no consanguineous family members, were enrolled in this study. This is the first study delineating the genetic causes of microcephaly in East Asia. The patients in this study showed the same clinical characteristics that were reported in previous studies, including infantile hypotonia and developmental delay. Epilepsy was observed in 65% of the patients, and approximately half of these patients showed an intractable clinical course. Additionally, movement disorders, behavioral problems, and autism spectrum disorder were noted, which were similar to the features reported in a recent study (Boonsawat et al., 2019). However, our study showed different genetic peculiarities from those reported previously for patients in the Middle East or Europe, in which autosomal recessive inheritance was frequent. In our study, 75% of the detected variants were autosomal dominant; only one sibling pair had an *SMC1A* gene variant with X-linked inheritance. These results suggest that the genetic spectrum of microcephaly may differ between ethnic groups, even if clinical characteristics are similar. Therefore, consideration of the racial background may be helpful to interpret WES results.

This study had several limitations. First, the number of patients in our study was small to determine the detection rate for genetic causes of microcephaly. Second, a functional study of detected VUSs was not performed. Although not classified as PVs or LPVs, some of the VUSs found in this study may be useful in determination of the genetic cause of microcephaly through additional studies to verify gene function. Additionally, we did not identify cases of somatic mosaicism and balanced translocation, which is difficult to confirm by WES or CMA. However, somatic mosaicism and balanced translocation have been rarely reported as genetic causes of microcephaly.

We conclude that conducting WES and CMA for patients with microcephaly is useful and time-/cost-effective for genetic diagnosis and treatment considerations. Unlike other studies, autosomal recessive microcephaly was rare, and autosomal dominant was the predominant mode of inheritance in Korean patients. This study confirms that microcephaly is a condition with genotypically and phenotypically heterogeneous causes. Interdisciplinary cooperation between molecular geneticists and clinicians is important to facilitate the elucidation of the underlying causes of microcephaly.

## Data Availability Statement

The datasets generated for this study can be found in the ClinVar: submission name as Korean microcephaly.

## Ethics Statement

Written informed consent was obtained from the individuals or legal guardian for the publication of any potentially identifiable images or data included in this article.

## Author Contributions

JeL, BK, and C-SK conceived and designed the study. JeL and JiL enrolled the participants and analyzed their clinical data. JP, CL, and AK analyzed the data of genetic evaluation. JiL and JP wrote the manuscript. WP and C-SK reviewed the analysis of genetic variants. JeL approved the submitted version of the manuscript. All authors contributed to the article and approved the submitted version.

## Conflict of Interest

C-SK, who previously belonged to Sungkyunkwan University School of Medicine during the period when this study was performed, was employed by the company Green Cross Genome from 2019. The remaining authors declare that the research was conducted in the absence of any commercial or financial relationships that could be construed as a potential conflict of interest.

## Supplementary Material

Supplementary Figure 1 Generation process of a candidate variant list using a four-step strategy.

Supplementary Table 1 List of genes included *in silico* gene panel for microcephaly.

Supplementary Table 2 Clinical phenotypes of all patients.

Supplementary Table 3 List of pathogenic variant, likely pathogenic variant, and variant of uncertain significance with *in-silico* analysis for microcephaly phenotype genes.
