# Supplementary figures and images for: Genomic Analysis of Korean Patient With Microcephaly
Source: Front Genet. 2021 Jan 28;11:543528. doi: 10.3389/fgene.2020.543528 (PMC7876370; doi:10.3389/fgene.2020.543528)

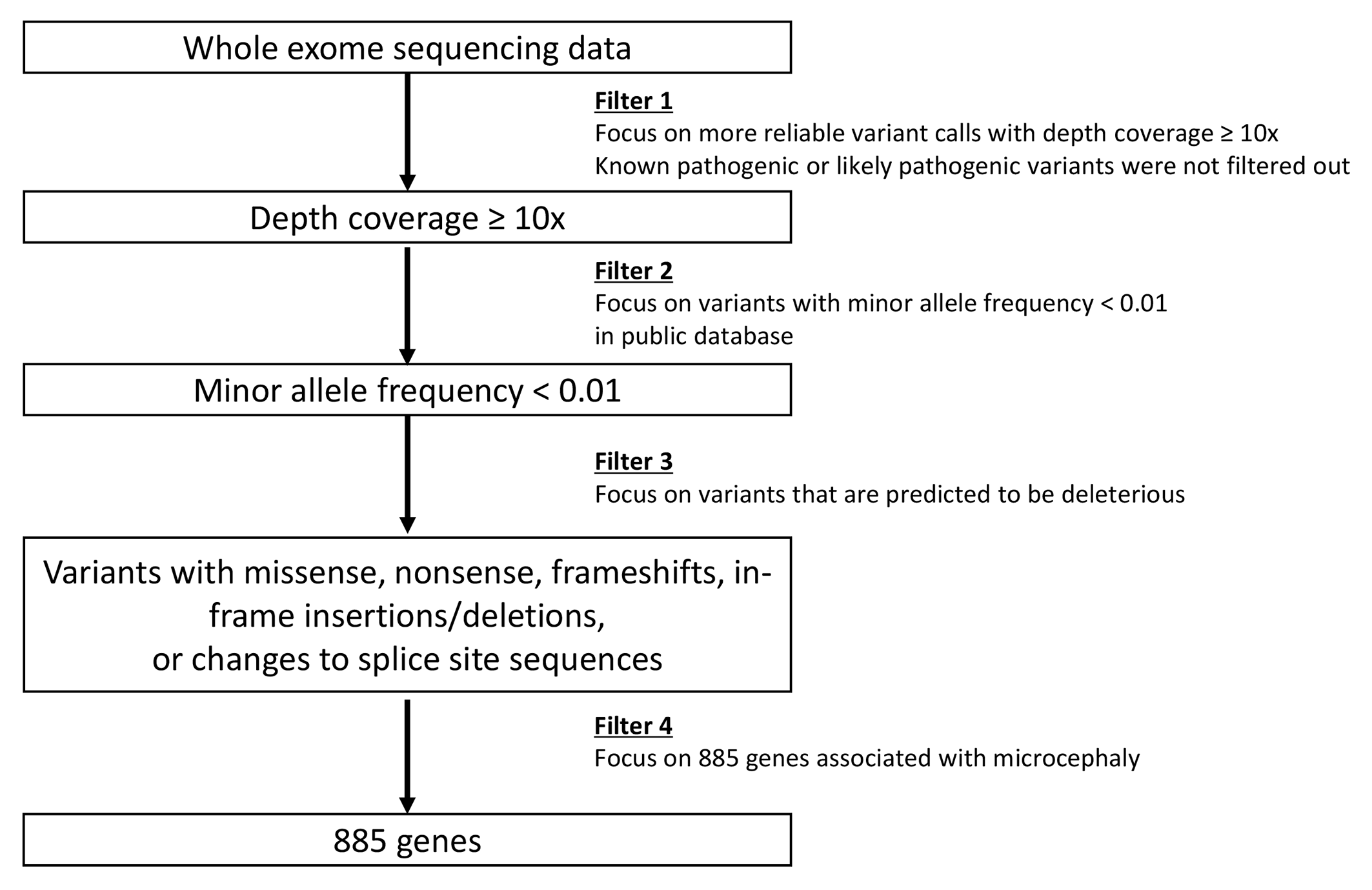

Supplement: Supplementary Figure 1 — Generation process of a candidate variant list using a four-step strategy. [file Image_1.TIF]
